# Artificial intelligence aided design of peptides with custom secondary structure motifs and reduced amino acid alphabets

**DOI:** 10.64898/2026.04.29.721096

**Published:** 2026-05-01

**Authors:** Sean M. Brown, Ashley B. Cohen, Scott N. Dean

**Affiliations:** 1 National Academies of Sciences, Engineering and Medicine, National Research Council Research Associateship, Program at US Naval Research Laboratory, Washington, DC, USA; 2 Center for Bio/Molecular Science and Engineering, US Naval Research Laboratory, Washington, DC, USA

**Keywords:** generative protein design, reduced amino acid alphabet, synthetic biology, artificial intelligence

## Abstract

Proteins are highly diverse functional polymers where the specific sequence of amino acids, selected from a standard genetically-encoded alphabet of twenty (C20), determines the structure and ultimately the function of the resulting folded protein. This standard alphabet has been identified to be non-randomly distributed in physicochemical properties crucial to both structure-formation and function, often referred to as coverage theory. While machine learning models have drastically improved protein structure prediction, protein design has yet to have similar development. Here we therefore bridge contemporary biological theory with recent advancements in artificial intelligence (AI) to develop and evaluate a generative AI protein design model, trained on hundreds of thousands of proteins within the RSCB PDB, for custom secondary structure motifs using reduced amino acid alphabets. Results indicate an overall success in designing novel proteins with desired secondary structure motifs for a broad range of amino acid alphabets. Interestingly this tool often captures the full three-dimensional tertiary structure of a target protein despite training only on physicochemical sequence space and DSSP secondary structure. The development of this model advances research across multiple disciplines, from general scientific AI/ML architecture development to protein design for biotechnology, astrobiology, and early-Earth evolutionary biology.

## Introduction

All life on Earth since the last universal common ancestor (LUCA) has constructed metabolism primarily as a network of genetically encoded proteins. Proteins are highly diverse functional polymers composed of an amino acid sequence ‘selected’ from a standard 20-member genetically-encoded alphabet (C20). In 1972, Christian Anfinsen was awarded the Nobel Prize in Chemistry for demonstrating that a protein’s primary sequence (i.e., the specific order of amino acids) determines how a linear polymer folds into a three-dimensional conformation, ultimately defining a protein’s function.^1^

Since then, it has been identified that the alphabet of 20 genetically encoded amino acids exhibits a statistically nonrandom distribution, or *coverage,* in van der Waals’ volume (size), and hydrophobicity.^2–6^ Both size and hydrophobicity are critical to the formation of protein structure: hydrophobic collapse is widely accepted as an underlying principle,^7–9^ even the driver,^10^ of protein folding, while volume determines the steric constraints available to structure formation. Coverage is defined by the combination of the range of descriptors measured for a set of amino acids, and the evenness with which they are distributed across such range (see Fig. SI.2). While range allows for chemical diversity, evenness minimizes the phenotypic jump as one amino acid substitutes for another in any given sequence for any ‘desired’ three-dimensional structure. Recent work has even applied coverage theory to successfully design, synthesize, and characterize xeno amino acids sets with familiar secondary structures such as α-helices.^6^ We therefore sought to determine if these coverage-theory-grounded design principles could be leveraged to design custom structural motifs using reduced amino acids more readily.

Due to recent advances in computational biology, research progress in canonical protein design has drastically accelerated over the last half decade. While *de novo* protein design has traditionally leveraged the reduction of sequence and feature space (e.g., Hecht binary patterning^11^), the use of artificial intelligence (e.g., ESM3^12^) to target a desired structure or function has emerged as the favored contemporary strategy. Recently developed models, largely with transformer architectures, are significantly more powerful than superannuated design methods.^13,14^ These models have proven capable of producing a wide variety of proteins with experimentally verified activity. For example, ProGen3 is a generative large language model that generates *de novo* protein sequences with similar properties to their naturally occurring analogs.^15^ Others, like ESM, can be utilized to simultaneously predict the structure and function of proteins via high quality embeddings.^12^ Additional critical developments here include protein generative diffusion models (e.g., RFdiffusion) for protein design^16^ and hybrid multimodal architectures that incorporate protein structure reinforcement learning to improve protein property predictions.^17^ These approaches enable the design of proteins with sequences and structures that are entirely novel compared to those found in nature. Recently, this expanded design space has been further extended by other newly developed computational tools, such as RareFold, which was developed for structure prediction and design of proteins containing xeno amino acids.^18^

**Figure 1.**
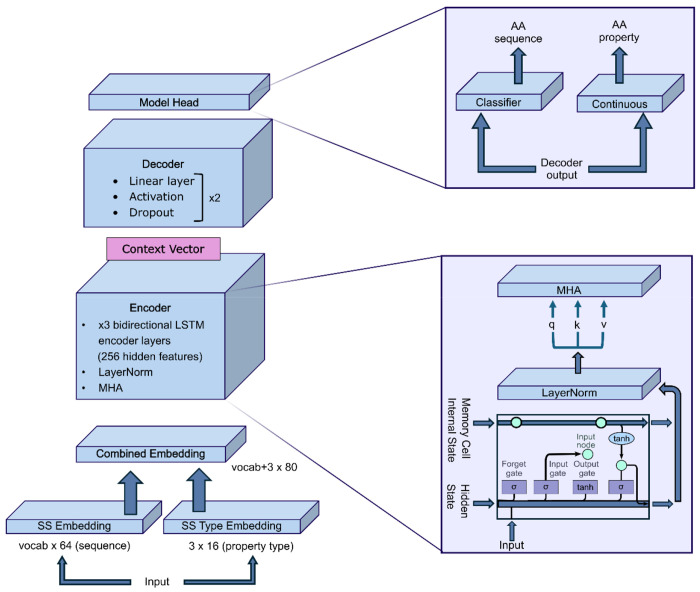
Major components of the bLSTMa encoder-decoder model architecture. Detailed architectures of the Encoder block, primarily made up of LSTM encoder layers and multi-head self-attention (bottom right) and model head, where the Decoder output is separately fed through a classifier and continuous value sequence to predict sequences and their associated properties (top right).

In contribution to this rapidly expanding protein design field, we developed and evaluated a generative artificial intelligence model (Fig. 1) to design novel proteins with custom secondary structure motifs. Our model, which consists of a bidirectional Long Short-Term Memory architecture with multi-head self-attention (bLSTMa), was trained on a large, comprehensive dataset of secondary structure assignments (Dictionary of Secondary Structure in Proteins^19^, DSSP) and associated physicochemical properties (van der Waals volume, logD, and formal charge at pH 7.0) from extracted proteins found in the RSCB Protein Databank (PDB).

Utilizing the dataset of over two-hundred thousand DSSP-assigned protein structures within the RSCB PDB and the calculated physicochemical properties at each residue, we designed and evaluated 29,387 sequences of proteins across 313 different amino acid alphabets to adopt numerous secondary structural motifs deriving from 101 target PDB structures. We evaluate the performance of our model by measuring accuracy metrics across various (i) protein lengths (ii) alphabet sizes (iii) structural diversity (iv) alphabet members and (v) three-dimensional structural propensity. Overall, results reveal that our model is generally successful in designing novel proteins with desired secondary structure motifs for a broad range of amino acid alphabets. Interestingly, many designs successfully retained the predicted targeted protein’s threedimensional tertiary structure using reduced amino acid alphabets, despite the lack of exposure to precise three-dimensional information during training.

## Results

The alphabets used in this study derive from a comprehensive review of various reduced amino acid alphabets.^20^ This collection of alphabets comprises unique combinations of ~86K alphabets resulting from 74 alphabet reduction methods. We then filtered this library to 312 alphabets by stratified sampling.

**Figure 2.**
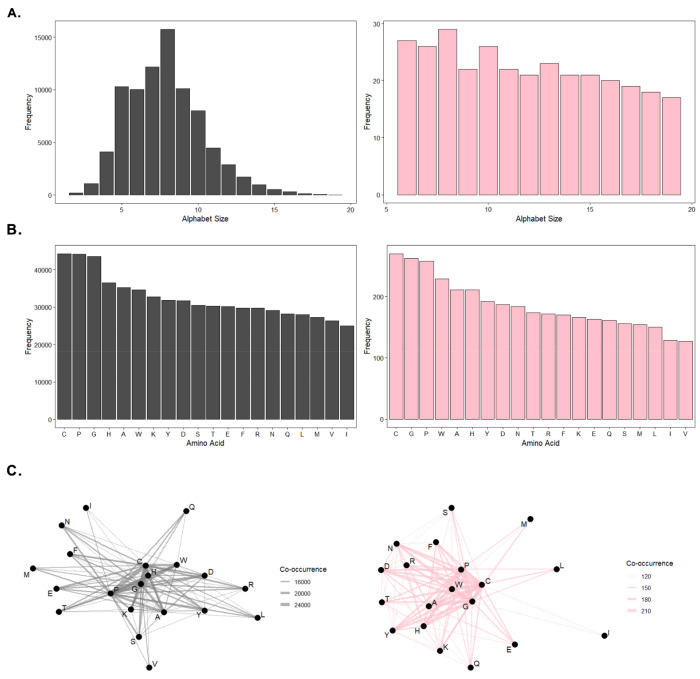
Background and subset alphabets characterization. **A.** distribution of background alphabets in terms of alphabet size for both the background ~83k (grey) and subset ~300 (pink) alphabets. **B.** distribution of background alphabets in terms of amino acid composition for both the background (grey) and subset (pink) alphabets. **C.** amino acid co-occurrence network for background alphabets for both the background (grey) and subset (pink) alphabets.

To establish a baseline, we first measured background distributions of the amino acid alphabets used in this study. Figure 2A. shows the distribution of unique reduced amino acid alphabets by alphabet size, amino acid composition, and co-occurrence. We observe that the size of the alphabets identified in previous work^20^ are right-tailed-normally distributed around a mean of 9.15 AAs and a standard deviation of 5.12 AAs. As expected, after filtering, the frequency of amino acids shifts to a uniform distribution (ranging from 6-19 AAs, x = 11.94 AAs, and σ = 4.00 AAs). Furthermore, Figure 2B. demonstrates that this uniformity did not come at the expense of altering the background amino acid frequency among these alphabets (which is also relatively uniform). We do observe a shift however in the filtered amino acid co-occurrence compared to the initial alphabet library (Fig. 2C.).

To develop a method that accurately generates amino acid sequences with desired secondary structure motifs, we trained our model on a diverse, non-redundant dataset of secondary structure assignments (DSSP) and associated physicochemical properties (van der Waals volume, logD, and formal charge at pH 7.0) extracted from >200K proteins within the RSCB PDB. Sequences in our non-redundant, clustered training dataset of 92,752 representative sequences had a minimum and maximum sequence length of 10 and 300, respectively, and selection of cluster representatives resulted in 1189 final target PDB structures. Of these, we obtained structures for a random selection of 101 PDB structures.

**Figure 3.**
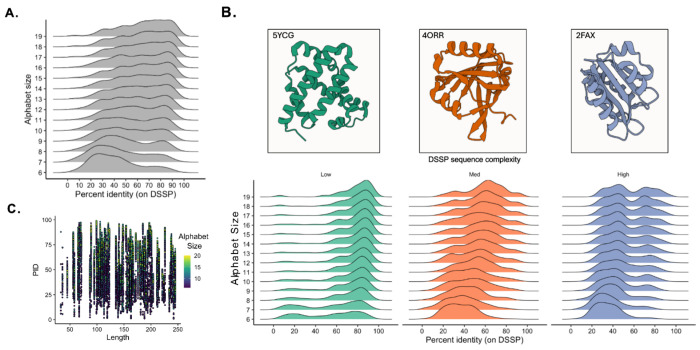
Model accuracy in terms of predicted DSSP secondary structure. **A.** DSSP sequence percent identity of predicted sequences to target, input, secondary structure. **B.** Example target structures of low (green) medium (orange) and high (blue) structure complexity proteins (top) displayed in ribbon format. Histograms (bottom) of results for DSSP sequence percent identity of predicted sequences to target, input, secondary structures for low- (green) medium- (orange) and high- (blue) complexity protein targets. **C.** DSSP sequence percent identity for predicted sequences across protein sequence length and alphabet size.

In order to evaluate the accuracy of the model in terms of predicted DSSP secondary structure, we calculated the percent identity (PID) of predicted sequences to the target secondary structure. After the model generates a sequence, we then predict secondary structure using AlphaFold2 (AF2), given the impracticality of synthesizing and characterizing thousands of predicted sequences. The model displays varying degrees of accuracy for generating reduced amino acid alphabet designs depending on the size of the alphabet (Fig. 3A). Generally, larger alphabets perform better in terms of PID compared to smaller alphabets (mean PID for size 19, 10, and 6 alphabets equals 66%, 54%, and 42%, respectively). We do observe a sharp decline in performance (shifting from a platykurtic to a right-skewed distribution) around alphabets size < 9. It’s important to note however that although the average PID decreased with alphabet size, the maximum did not. In other words, while it may be significantly less abundant, accurate DSSP designs can be found for all alphabets tested.

The accuracy of the generated sequences does appear to depend on the overall complexity of the target structure (Fig. 3B). Low-complexity structures such as primarily helix are therefore more abundant when using reduced amino acid alphabets compared to higher complexity structures (e.g., a helix-coil-sheet-coil-sheet-coil-helix motif). As structural complexity increases, we generally observe a shift from a left-tailed distribution (low complexity, mean PID = 68%) to a relatively platykurtic distribution (medium complexity, mean PID = 50%), to a bimodal or righttailed distribution (high complexity, mean 1 PID = 38%, mean 2 PID = 73%). Although far less common than designs with low-complexity structures, we do achieve high PID designs with high-complexity structure using alphabets as small as 6 amino acids. Additionally, we tested model accuracy as a function of protein sequence length. Model accuracy as a function of target DSSP PID does not depend on the generated protein’s amino acid sequence length, demonstrating that our method works equally well with a broad size-range of target structures (Fig. 3C).

To further investigate the features governing design success, we analyzed the relationship between DSSP sequence properties and PID. An XGBoost model trained on sequence-derived features was able to accurately predict whether a design would have high or low PID, achieving a receiver operating characteristic area under the curve (ROC-AUC) of 0.98 (Fig. SI.1B). This indicates that design accuracy is strongly determined by the properties of the target secondary structure sequence. Feature importance analysis of this model revealed that properties such as sequence entropy and transition frequencies were highly predictive of accuracy (Fig. SI.1C), suggesting that complex input DSSP sequences are less likely to yield high-PID designs.

**Figure 4.**
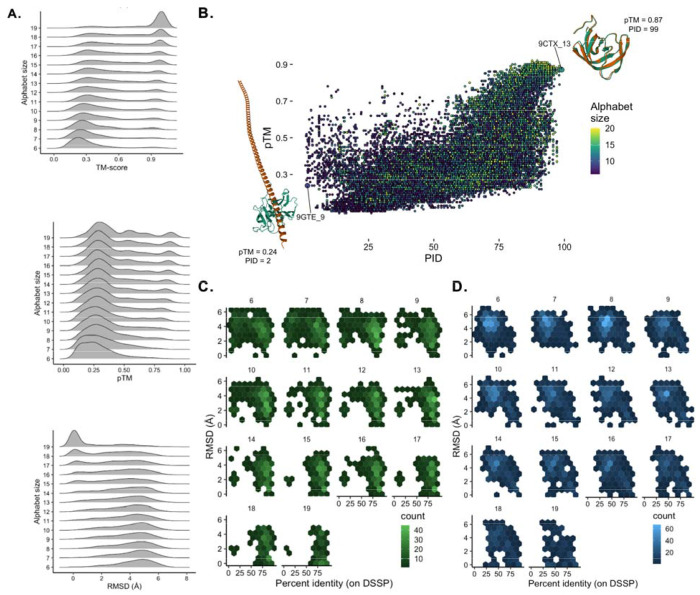
Model accuracy in terms of target tertiary structure. **A.** Accuracy for various amino acid alphabet sizes (6-19) measured by AF2 TM-score (left), pTM (middle), and RMSD (right). **B.** Percent identity (PID) and pTM scores across various alphabet sizes (6-19). Lowest (by pTM and PID – 9GTE_9) and highest (9CTX_13) accuracy to target structures (green – target; orange – AF2 predicted model output sequence) shown in bottom left and top right resp. **C-D.** RMSD versus DSSP percent identity correlation for low (**C.** - green) and high (**D.** - blue) structur**e** complexity sequences.

Beyond the overall predictive accuracy of target secondary structure, we additionally investigated if our model potentially learned underlying principles which govern the threedimensional folding of amino acid chains. To do so, we measured the extent to which the threedimensional (tertiary) structure of target proteins is preserved in our model’s output design constructs. Remarkably, despite having only been trained on the one-dimensional information of secondary structure sequence motifs and associated physicochemical properties, the model’s output appears to unintentionally capture the precise tertiary structure of the PDB target, to varying alphabet-size dependent accuracy (Fig. 4). This finding’s consistency with observed DSSP PID accuracy indicates that training merely on secondary structures and sequence-dependent physicochemical data appears to be sufficient for accurate three-dimensional design rather than relying on extracted patterns within life’s amino acid sequence-structure space (i.e., homology modeling – as in traditional models). As expected, this outcome appears to depend upon which amino acid alphabet is provided to our model. Specifically, we observe a clear relationship between amino acid alphabet size and three-dimensional structural accuracy where an increase in alphabet size typically improves both TM- and pTM-scores while also lowering the backbone RMSD (Fig. 4A). There is a noticeable shift in TM- and pTM- scores from a unimodal (high-accuracy) to bi- or multimodal distribution between 8-18 amino acid alphabets, before returning to a unimodal (poor-accuracy) distribution when using alphabets around <8 amino acids in length. This shift is also observed for RMSD albeit to a lesser extent than TM- and pTM- scores. This finding suggests heavyweight models and immense datasets may not always be required, depending on the target sequence and complexity.

While these three metrics of three-dimensional accuracy generally concur, we do not observe as strong of a relationship for measures of three-dimensional accuracy, DSSP percent identity, and alphabet size (Fig. 4B). We also do not observe a strong correlation between PID and pTM (R^2^ = 0.66). Furthermore, while the more accurate predictions were those with larger alphabets and the poor accuracy predictions were typically observed with smaller alphabets, the space between these two extremes is far more complex. For example, one of the most accurate constructs observed (9CTX_13; pTM = 0.87; PID = 99%) derives from a 13-mer alphabet; therefore underscoring how high-accuracy constructs (in both DSSP and three-dimensional structure as predicted by AF2) are still available to smaller alphabets. Moreover, Figure 4C–D shows that high-accuracy constructs (particularly for larger amino acid alphabets) also retained a relatively low RMSD to the three-dimensional structure of the original target in PDB. While this pattern is true for high-complexity sequences (Fig. 4D) it is, perhaps unsurprisingly, stronger for low complexity sequences (Fig. 4C). The probability of this phenomenon occurring for a more complex protein however would intuitively be lower than with a less complex structure (e.g., only alpha helix). One plausible explanation of this finding is that there are physical constraints on secondary structure patterns which result in only one structure for each secondary structure pattern. Alternatively, an equally likely explanation may be artifacts deriving from the training data for AF2 structure prediction; where the only available data of course was all of life’s proteins and therefore AF2 could not have learned other ways to form these three-dimensional patterns. At current, the shear lack of experimentally resolved constructs built with alphabet’s beyond C20 (N=1, Xeno Peptide P2^6^) obscures where the truth lies between these two explanations.

## Discussion

In this work we developed a bLSTMa machine learning model, trained on a large, diverse, non-redundant dataset of secondary structure assignments (DSSP) and associated physicochemical properties. This model can be used as a physicochemically-grounded protein design tool for any desired secondary structural motif and nearly any amino acid alphabet. To benchmark our model, we designed and evaluated many proteins comprising various canonical amino acid alphabets to adopt the same secondary structural motifs observed in 101 target PDB protein structures. Results reveal that this method is preponderantly able to design proteins successfully with any of a number of reduced amino acid alphabets based on fundamental physicochemical properties. The development of this tool advances research progress across multiple disciplines, including general scientific AI/ML architecture development, protein design for biotechnology, astrobiology, and early-Earth evolutionary biology.

### Implications for artificial intelligence and machine learning.

As the field of biology-focused artificial intelligence continues to rapidly grow, vision, language, and multimodal AI/ML models have become larger and more complex, often having hundreds of millions to tens of billions of trainable parameters.^18^ While larger models often exhibit superior performance, in practice, they are rarely used given the enormous computational resources required (ibid.). By contrast, to sufficiently train our 5.1 million parameter model, 70% of data entries need to be seen. Even with small batching (8-10 entries), training can be completed in 50 epochs or 2 hours wall time, overall achieving a relatively low computational training cost. Furthermore, we demonstrate that our model successfully generates protein sequences with numerous amino acid alphabets at state-of-the-art predicted performance using a simple LSTM encoder-decoder architecture with a modest number of trainable parameters and small vocabulary size. Our lightweight architecture therefore allows for greater user accessibility, more precise architecture refinement and scaling, faster training, and richer contextual embeddings. As such, we anticipate that a broader user-developer community will iteratively refine and improve upon this method (e.g., dead node pruning, adjusting the context window size, changing the number of encoder layers, etc.). Furthermore, the bLSTMa is trained on inherently more enriched embeddings compared to a standard LLM. Most LLMs feed a single unidirectional embedding through their architecture as a consequence of being generative transformers (i.e., autoregressive, single-direction, prediction of new tokens from prior tokens). The model used here instead concatenates bidirectional embeddings of secondary structure and physicochemical properties into a highly enriched combined contextual embedding. The low-loss and reduced training overhead of bidirectional embeddings has been demonstrated by the popularity and success of a similar approach: ProteinBERT.^19^ While quick and easy to use, ProteinBERT only predicts protein properties, while the model demonstrated here is for generative protein design.

### Implications for protein design.

Our approach advances protein design by establishing a first approximation of the minimum number of physicochemical descriptors needed to reliably generate protein sequences based solely on a desired sequence of secondary structural motifs. Rather than exhaustively including a large dataset of numerous physicochemical descriptors, we built our framework on one set of “essential” parameters through optimality and evolutionary amino acid alphabet coverage theories. This simple, physicochemically-grounded approach keeps the model’s vocabulary size small, which directly determines the size of the model weights’ last tensor dimension. A small tensor size not only decreases training time, but also drastically reduces the overhead memory requirements to train the model. This is particularly interesting when compared to protein language models like ProGen2, which pre-train using all available taxonomy, function, and ontogeny ids associated with proteins to create prepended conditional “tags”, resulting in an enormous vocabulary.

The novelty of this approach is not limited to just life’s amino acids. Very few tools exist for xeno peptide and protein research^6^ apart from novel approaches like RareFold^21^ given the lack of data beyond the only experimentally verified xeno peptide P2.^6^ Because our bLSTMa was trained using position-dependent physicochemical properties and secondary structure assignments instead of amino acids themselves, our model is, in theory, compatible with any amino acid alphabet, including xeno amino acid alphabets. The accuracy of structures predicted from xeno amino acid alphabets however is yet to be determined because of the fundamental lack of synthesized xeno peptides or proteins to date. In other words, it is uninformative to assess the performance of a polymer-class where the sample size equals one. The approach presented here therefore guides any synthetic biologist to efficiently design proteins or peptides to a high degree of confidence with any canonical amino acid alphabet while not excluding compatibility for xeno amino acid polymers. As this framework is adapted and built upon, it will become clearer how many and which physicochemical parameters are needed to most accurately distill the physics of protein secondary structure and to what extent additional physicochemistry leads to diminishing returns in training and prediction.

### Implications for astrobiology and evolutionary biology.

The method presented in this study additionally advances the fields of astrobiology and evolutionary biology. Our bLSTMa acts as a novel framework for scientists to design and test theorized primordial or xeno protein candidates. With the model presented here, and specifically with the ability to confidently design structures from reduced amino acid alphabets, we present a new foundation with which to probe evolutionary history of proteins prior to LUCA. Similarly, this approach informs how easily an alternative origin of life (bearing protein biochemistry) may emerge by enabling the beginnings to answer: could alternative amino acid alphabets form eerily similar proteins with entirely different building blocks?

## Conclusions

Our model trained on a limited subset of physicochemical parameters and our benchmarking protocol is proof-of-concept that a physics and chemistry-based approach can be used to design and generate proteins with various amino acid alphabets and desired secondary structures. Many other physicochemical parameters exist that may or may not improve the model’s ability to design peptides or proteins. The physicochemical combinatorial space is simply too vast to be completely explored. It is therefore a tractable near-future research direction to begin traversing that space by testing the extent to which other physicochemical properties contribute to the formation of fundamental protein secondary structure. Additionally, it remains unknown if and to what extent a similar small-parameter model could successfully generate structurally and functionally novel protein and peptide sequences.

## Methods

### Library Curation.

Our machine learning model was trained on all proteins within the RCSB PDB (www.rcsb.org) protein-only group (as of 17DEC2025, N=202,134). For each protein, the DSSP^19,22^ sequence was extracted if available, or calculated, and a sequence of physicochemical descriptors (van der Waals volume, logD, and formal charge at pH 7.0; Table 1) were assigned to each residue in each sequence. Each physicochemical descriptor was calculated using ChemAxon JChem, https://www.chemaxon.com, (see Mayer-Bacon and Yirk^23^ to see in-depth how to calculate these descriptors).

### Design Space Selection.

We down-selected from the 82,795 distinct reduced canonical amino acid alphabets in Liang et al.^20^ to 312 by performing stratified sampling after removing alphabets of size 5 or smaller, grouping by alphabet size and method (as defined by Liang et al.) and randomly selecting one per group (Table SI.1). To reduce the combinatorially enormous set of target proteins to test while retaining maximum sequence diversity, we clustered 2,612,149 individual DSSP sequences into 92,752 representative sequences using CD-HIT^24^ with cluster threshold set to 0.8. From these clustered sequences, we removed clusters with fewer than 10 members and selected the designated representatives of each cluster, followed by removal of sequences longer than 300 amino acids, yielding 1,189 final sequences. The full design space comprised 1,189 targets × 313 alphabets (312 reduced + 1 full) = 370,957 predicted sequences. Due to computational resource limitations, ColabFold^25^ structure predictions were completed for 101 of the 1,189 targets, yielding 29,387 unique (target, alphabet) combinations with fold confidence scores (pTM). Of these, 85 targets were evaluated across all 313 alphabets, while 16 targets had partial coverage due to ColabFold run failures. Structural alignment (RMSD and TM-score) was obtained for 29,728 of the 33,573 total predictions due to reference PDB or ColabFold file errors.

### Machine Learning Model.

Our model, implemented in PyTorch, has an encoder-decoder architecture featuring bidirectional LSTM layers and a multi-head self-attention mechanism (Fig. 1), totaling 5,065,280 trainable parameters. The combined embedding first passes through the encoder block, which consists of three bidirectional long short-term memory (LSTM) encoder layers with 256 hidden features, layer normalization and 8-head multi-head attention. The resulting context vector is then passed into the decoder block, which consists of 2 iterations of a linear layer, rectified linear unit (ReLU) activation function and dropout sequence.

The dataset was randomly split 70/20/10 into training, validation, and test sets, batched into groups of eight. DSSP sequences were tokenized into a vocabulary of 9 states (α-helix, 3- helix, π-helix, β-strand, bridge, turn, bend, polyproline, coil) and embedded as 64-dimensional vectors, concatenated with a 16-dimensional secondary structure type embedding (helix/sheet/coil), yielding an 80-dimensional combined input. The model was trained for 50 epochs using AdamW (learning rate = 0.001, weight decay = 0.01) with a cosine annealing warm restart schedule (T = 20, T_mult = 2). Temperature was linearly annealed from 2.0 to 0.5 over training to transition from exploratory to exploitative optimization. The loss function combined three terms: property MSE (a = 1.0), cross-entropy with teacher forcing (β = 0.2), and entropy regularization (γ = 0.01), with an additional L2 penalty on logits. Gradient clipping (max norm = 1.0) and mixed precision training were used for stability and efficiency. The classifier output layer weights were initialized with Xavier Uniform (gain = 0.1) and zero bias.

During inference, the model outputs logits over all 20 amino acids at each position. For reduced alphabet predictions, logits corresponding to disallowed amino acids are set to negative infinity before applying softmax, effectively restricting the output distribution to only the permitted alphabet. The amino acid with the highest probability at each position is then selected (greedy decoding).

### Structure Prediction and Validation.

Predicted protein sequences were folded using LocalColabFold (v1.5.5) with AlphaFold2 (v2.3.6), run locally with MMseqs2 (v15.6f452) for multiple sequence alignment, JAX (v0.4.23) with CUDA 11/cuDNN 8.6, and Python 3.10.13. The predicted template modeling score (pTM) was extracted from ColabFold output for each prediction as a measure of fold confidence. To assess structural accuracy, predicted structures were compared to experimentally resolved target structures from the RCSB PDB using TM-align, yielding RMSD and TM-score metrics.

### Performance Quantification.

To evaluate the range of target sequence complexity over which the design method is effective, we computed a composite complexity score for each DSSP sequence from three metrics: (1) Shannon entropy, measuring the diversity of secondary structure states in the sequence; (2) normalized transition frequency, measuring the rate of secondary structure type changes along the sequence normalized by maximum possible transitions; and (3) Simpson diversity index, measuring the evenness of secondary structure state usage. Each metric was normalized to the range [0, 1] (Shannon entropy was divided by log (9), the maximum entropy for 9 DSSP states) and the composite complexity score was computed as the equally weighted mean of the three metrics. Target sequences were grouped into three equally sized complexity categories (low, medium, and high) using quantile-based binning to examine the relationship between target complexity and design accuracy.

So as to quantify model accuracy, we measured the percent identity of predicted DSSP sequence to the target DSSP sequence (PID) across alphabet sizes and target complexities. PID was calculated using pairwise sequence alignment via the Biostrings (v2.74.1) and pwalign (v1.2.0) R packages. As a metric for three-dimensional accuracy, we additionally measured the RMSD of predicted output structures to the experimentally resolved target structures found in the RCSB PDB.

### XGBoost Classifier.

To identify sequence-level features predictive of high design accuracy, a XGBoost binary classifier was trained to distinguish between high (PID > 80%) and low (PID < 80%) design outcomes. Input features were pulled from the predicted protein sequence and included peptide-level physicochemical descriptors (aliphatic index, GRAVY hydrophobicity index, molecular weight, net charge, isoelectric point, FASGAI alpha-turn propensity, flexibility index, polarizability, charge composition, and solvent accessibility) computed using functions from Peptides (v2.4.6 )^26^ and protr (v1.7–4)^27^ R packages, as well as mono-, di-, and tri-peptide composition frequencies. The model was implemented using the tidymodels framework (v1.3.0) in R (v4.4.3) with the XGBoost engine. The dataset was split 80/20 into training and test sets, stratified by PID class. Six hyperparameters (tree depth, minimum node size, loss reduction, sample size, number of features per split, and learning rate) were tuned via Latin hypercube sampling over 300 combinations with 3-fold cross-validation, optimizing ROC-AUC. The best hyperparameter combination was selected and the final model was evaluated on the held-out test set.

## Supplementary Material

Supplement 1

## Figures and Tables

**Table 1.** Physicochemical value for each genetically encoded amino acid at pH 7.0.

Amino Acid (1-Letter)	Van Der Waals Volume (Å^3^)	JChem LogD	Formal Charge
A	86.4	−1.34	0
C	104.9	−1.29	0
D	118.6	−1.98	−1
E	135.9	1.70	−1
F	159.0	0.31	0
G	69.1	−1.91	0
H	131.7	−2.11	0
I	138.3	−0.01	0
K	149.3	−1.48	+1
L	138.3	−0.09	0
M	139.5	−0.69	0
N	120.8	−2.79	0
P	108.6	−1.07	0
Q	138.1	−2.50	0
R	168.6	−2.54	+1
S	95.2	−2.39	0
T	112.5	−1.97	0
V	121	−0.46	0
W	178.7	0.41	0
Y	167.8	0.01	0

## Data Availability

All data needed to evaluate the conclusions in the paper are present in the paper, the Supplementary Information. Any additional data is available by request.

## References

[1] Anfinsen Christian B.. The Nobel Prize in Chemistry. (1972).

[2] Ilardo M., Meringer M., Freeland S., Rasulev B. & Cleaves H. J. Extraordinarily adaptive properties of the genetically encoded amino acids. Sci. Rep. 5, 9414 (2015). 25802223 10.1038/srep09414 PMC4371090

[3] Mayer-Bacon C., Meringer M., Havel R., Aponte J. C. & Freeland S. A Closer Look at Non-random Patterns Within Chemistry Space for a Smaller, Earlier Amino Acid Alphabet. J. Mol. Evol. 90, 307–323 (2022). 35666290 10.1007/s00239-022-10061-5

[4] Brown S. M., Vorácek V. & Freeland S. What Would an Alien Amino Acid Alphabet Look Like and Why? Astrobiology 23, 536–549 (2023). 37022727 10.1089/ast.2022.0107

[5] Brown S. M., Mayer-Bacon C. & Freeland S. Xeno Amino Acids: A Look into Biochemistry as We Do Not Know It. Life Basel Switz. 13, 2281 (2023). 10.3390/life13122281 PMC10744825 38137883

[6] Brown S. M. Xeno amino acid alphabets form peptides with familiar secondary structure. 2026.01.28.701962 Preprint at 10.64898/2026.01.28.701962 (2026).

[7] Kauzmann W. Some factors in the interpretation of protein denaturation. Adv. Protein Chem. 14, 1–63 (1959). 14404936 10.1016/s0065-3233(08)60608-7

[8] Agashe V. R., Shastry M. C. & Udgaonkar J. B. Initial hydrophobic collapse in the folding of barstar. Nature 377, 754–757 (1995). 7477269 10.1038/377754a0

[9] Robson B. & Pain R. H. Analysis of the code relating sequence to conformation in proteins: Possible implications for the mechanism of formation of helical regions. J. Mol. Biol. 58, 237–257 (1971). 5088928 10.1016/0022-2836(71)90243-9

[10] Salamatova E. Hydrophobic Collapse in N-Methylacetamide–Water Mixtures. J. Phys. Chem. A 122, 2468–2478 (2018). 29425450 10.1021/acs.jpca.8b00276 PMC6028151

[11] Bradley L. H., Wei Y., Thumfort P., Wurth C. & Hecht M. H. Protein Design by Binary Patterning of Polar and Nonpolar Amino Acids. in Protein Engineering Protocols (eds. Arndt K. M. & Müller K. M.) 155–166 (Humana Press, Totowa, NJ, 2007). doi:10.1385/1-59745-187-8:155. 17041264

[12] Hayes T. Simulating 500 million years of evolution with a language model. Science 387, 850–858 (2025). 39818825 10.1126/science.ads0018

[13] Huang P.-S., Boyken S. E. & Baker D. The coming of age of de novo protein design. Nature 537, 320–327 (2016). 27629638 10.1038/nature19946

[14] Kortemme T. De novo protein design—From new structures to programmable functions. Cell 187, 526–544 (2024). 38306980 10.1016/j.cell.2023.12.028 PMC10990048

[15] Bhatnagar A. Scaling Unlocks Broader Generation and Deeper Functional Understanding of Proteins. 2025.04.15.649055 Preprint at 10.1101/2025.04.15.649055 (2025).

[16] Butcher J. De novo Design of All-atom Biomolecular Interactions with RFdiffusion3. 2025.09.18.676967 Preprint at 10.1101/2025.09.18.676967 (2025).

[17] Abramson J. Accurate structure prediction of biomolecular interactions with AlphaFold 3. Nature 630, 493–500 (2024). 38718835 10.1038/s41586-024-07487-w PMC11168924

[18] Li Q. RareFold: Structure prediction and design of proteins with noncanonical amino acids. 2025.05.19.654846 Preprint at 10.1101/2025.05.19.654846 (2025).

[19] Hekkelman M. L., Salmoral D. Á., Perrakis A. & Joosten R. P. DSSP 4: FAIR annotation of protein secondary structure. Protein Sci. Publ. Protein Soc. 34, e70208 (2025). 10.1002/pro.70208 PMC12268231 40671631

[20] Liang Y. Research progress of reduced amino acid alphabets in protein analysis and prediction. Comput. Struct. Biotechnol. J. 20, 3503–3510 (2022). 35860409 10.1016/j.csbj.2022.07.001 PMC9284397

[21] Li Q., Daumiller D. & Bryant P. RareFold: Structure prediction and design of proteins with noncanonical amino acids. 2025.05.19.654846 Preprint at 10.1101/2025.05.19.654846 (2025).

[22] Kabsch W. & Sander C. Dictionary of protein secondary structure: pattern recognition of hydrogen-bonded and geometrical features. Biopolymers 22, 2577–2637 (1983). 6667333 10.1002/bip.360221211

[23] Mayer-Bacon C. & Yirik M. A. Curation of Computational Chemical Libraries Demonstrated with Alpha-Amino Acids. J. Vis. Exp. JoVE 10.3791/63632 (2022) doi:10.3791/63632. 35499348

[24] Fu L., Niu B., Zhu Z., Wu S. & Li W. CD-HIT: accelerated for clustering the nextgeneration sequencing data. Bioinformatics 28, 3150–3152 (2012). 23060610 10.1093/bioinformatics/bts565 PMC3516142

[25] Kim G. Easy and accurate protein structure prediction using ColabFold. Nat. Protoc. 20, 620–642 (2025). 39402428 10.1038/s41596-024-01060-5

[26] Osorio D., Rondón-Villarreal P. & Torres R. Peptides: A Package for Data Mining of Antimicrobial Peptides. R J. 7, 4–14 (2015).

[27] Xiao N., Cao D.-S., Zhu M.-F. & Xu Q.-S. protr/ProtrWeb: R package and web server for generating various numerical representation schemes of protein sequences. Bioinformatics 31, 1857–1859 (2015). 25619996 10.1093/bioinformatics/btv042

