# Supplementary material for: Artificial intelligence aided design of peptides with custom secondary structure motifs and reduced amino acid alphabets
Source: bioRxiv. 2026 Jul 7:2026.04.29.721096. Originally published 2026 May 1. Preprint. [Version 2] doi: 10.64898/2026.04.29.721096 (PMC13142411; doi:10.64898/2026.04.29.721096)
Supplement: Supplement 1 [file media-1.pdf]

SUPPLEMENTARY INFORMATION

FOR

Machine Learning Aided Design of Peptides with Custom Secondary Structure Motifs and Reduced Amino Acid Alphabets

Sean M. Brown [1] Ashley B. Cohen [1] Scott N. Dean [2*]

[1]National Academies of Sciences, Engineering and Medicine, National Research Council Research Associateship Program at US Naval Research Laboratory, Washington, DC, USA
[2]Center for Bio/Molecular Science and Engineering, US Naval Research Laboratory, Washington, DC, USA

[*]**Correspondence to:** Scott N. Dean, Center for Bio/Molecular Science and Engineering, U.S. Naval Research Laboratory, 4555 Overlook Avenue – SW, Bldg. 30 / Code 6900, Washington, DC 20375, USA. Email: scott.n.dean.civ@us.navy.mil

DISTRIBUTION STATEMENT A. Approved for public release: distribution is unlimited.

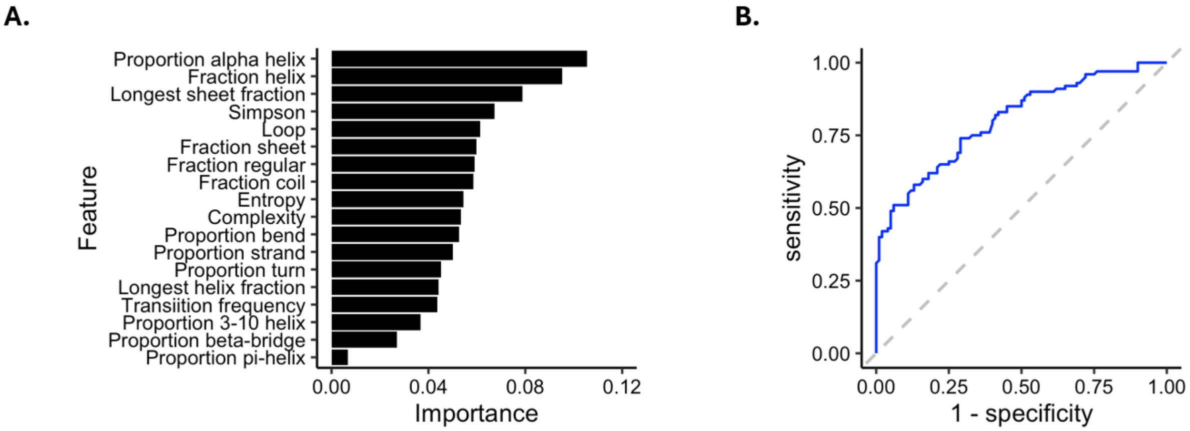

Figure SI.1. DSSP percent identity prediction using sequence-based features. A. Receiver operating characteristic curve (ROC) of the best-performing classifier. ROC-AUC = 0.98. **B.** Top 18 features ranked by importance for the Random Forest classifier.

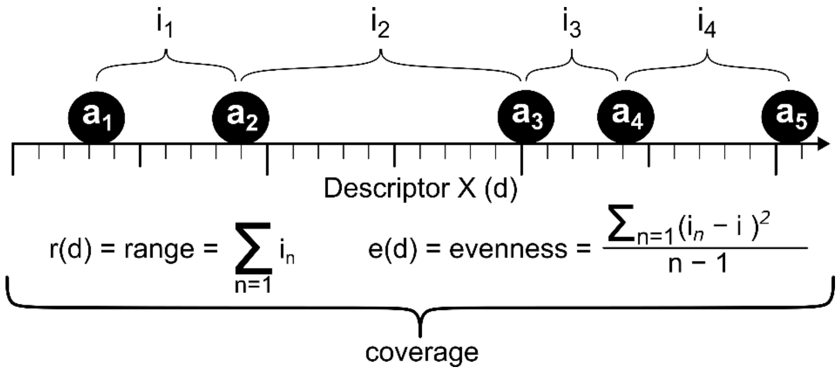

Figure SI.2. Physicochemical coverage - definition of range and evenness. For a given chemical descriptor such as van der Waals volume, "coverage" joins two statistics to characterize a set of amino acids. These statistics are shown for an example set of five amino acids ($a_1 \ldots a_5$) with four corresponding intervals ($i_1 \ldots i_4$) measured in terms of the hypothetical quantitative 'descriptor x' (*d*). Evenness (*e*) is the sample variance of the intervals between amino acids (*i*); Range (*r*) is the sum of these intervals ($\sum i_{1\ldots4}$); "Coverage" is therefore the combination of range AND evenness for any given physicochemical descriptor.

Table SI.1 Stratified Reduced Amino Acid Alphabets

Method	Size	Reduced Code	Combination
AIS	6	SWGA-DRHQYNE-KLVIF-C-PM-T	W-D-L-C-M-T
AIS	7	SQLGE-DRP-KT-W-HN-C-VIMAFY	G-R-K-W-H-C-Y
AIS	8	DKP-SRGFNE-WH-C-Q-LVMAY-I-T	P-S-W-C-Q-A-I-T
AIS	9	SGA-DP-RWHYN-KE-C-Q-LT-IV-MF	A-P-N-E-C-Q-L-V-M
BLOSUM40	5	AGPST-RNDQEHK-C-ILMFYV-W	G-D-C-M-W
BLOSUM40	6	AGPST-RNDQEK-C-H-ILMFYV-W	A-E-C-H-M-W
BLOSUM40	7	ANDGST-RQEK-C-H-ILMFYV-P-W	G-Q-C-H-L-P-W
BLOSUM40	8	ANDGST-RQEK-C-H-ILMV-FY-P-W	G-Q-C-H-M-Y-P-W
BLOSUM40	9	AGST-RQEK-ND-C-H-ILMV-FY-P-W	T-Q-D-C-H-I-F-P-W
BLOSUM40	10	AGST-RK-ND-C-QE-H-ILMV-FY-P-W	S-R-D-C-E-H-L-Y-P-W
BLOSUM40	11	AST-RK-ND-C-QE-G-H-ILMV-FY-P-W	A-R-N-C-E-G-H-V-F-P-W
BLOSUM40	12	AST-RK-ND-C-QE-G-H-IV-LM-FY-P-W	S-K-N-C-Q-G-H-V-L-Y-P-W
BLOSUM40	13	AST-RK-N-D-C-QE-G-H-IV-LM-FY-P-W	A-K-N-D-C-Q-G-H-V-M-F-P-W
BLOSUM40	14	AST-RK-N-D-C-Q-E-G-H-IV-LM-FY-P-W	S-R-N-D-C-Q-E-G-H-I-M-F-P-W
BLOSUM40	15	A-RK-N-D-C-Q-E-G-H-IV-LM-FY-P-ST-W	A-R-N-D-C-Q-E-G-H-V-L-F-P-T-W
BLOSUM40	16	A-RK-N-D-C-Q-E-G-H-IV-LM-F-P-ST-W-Y	A-K-N-D-C-Q-E-G-H-V-L-F-P-S-W-Y
BLOSUM40	17	A-R-N-D-C-Q-E-G-H-IV-LM-K-F-P-ST-W-Y	A-R-N-D-C-Q-E-G-H-V-L-K-F-P-T-W-Y
BLOSUM40	18	A-R-N-D-C-Q-E-G-H-IV-LM-K-F-P-S-T-W-Y	A-R-N-D-C-Q-E-G-H-I-M-K-F-P-S-T-W-Y
BLOSUM40	19	A-R-N-D-C-Q-E-G-H-IV-L-K-M-F-P-S-T-W-Y	A-R-N-D-C-Q-E-G-H-I-L-K-M-F-P-S-T-W-Y
BLOSUM50	5	IMVL-FWY-G-PCAST-NHQEDRK	V-W-G-P-H
BLOSUM50	6	LVIM-AGST-PHC-FYW-EDNQ-KR	V-S-C-Y-N-K
BLOSUM50	7	IMVL-FWY-G-P-CAST-NHQED-RK	V-Y-G-P-A-Q-R
BLOSUM50	8	LVIMC-AG-ST-P-FYW-EDNQ-KR-H	M-G-S-P-F-E-K-H
BLOSUM50	9	IMV-L-FWY-G-P-C-AST-NHQED-RK	M-L-F-G-P-C-A-D-K
BLOSUM50	10	IMV-L-FWY-G-P-C-A-STNH-QERK-D	I-L-F-G-P-C-A-T-E-D
BLOSUM50	11	IMV-L-FWY-G-P-C-A-STNH-QRK-E-D	V-L-F-G-P-C-A-S-R-E-D
BLOSUM50	12	LVIM-C-A-G-ST-P-FY-W-EQ-DN-KR-H	I-C-A-G-T-P-Y-W-E-N-K-H
BLOSUM50	13	IMV-L-F-WY-G-P-C-A-ST-N-HQRK-E-D	M-L-F-W-G-P-C-A-S-N-K-E-D
BLOSUM50	14	IMV-L-F-WY-G-P-C-A-S-T-N-HQRK-E-D	V-L-F-Y-G-P-C-A-S-T-N-K-E-D
BLOSUM50	15	IMV-L-F-WY-G-P-C-A-S-T-N-H-QRK-E-D	M-L-F-W-G-P-C-A-S-T-N-H-K-E-D
BLOSUM50	16	IMV-L-F-W-Y-G-P-C-A-S-T-N-H-QRK-E-D	V-L-F-W-Y-G-P-C-A-S-T-N-H-R-E-D
BLOSUM50	18	LM-VI-C-A-G-S-T-P-F-Y-W-E-D-N-Q-K-R-H	M-I-C-A-G-S-T-P-F-Y-W-E-D-N-Q-K-R-H
BLOSUM62	5	FWYH-MILV-CATSP-G-NQDERK	Y-L-A-G-K
BLOSUM62	6	FWYH-MILV-CATS-P-G-NQDERK	Y-I-A-P-G-Q
BLOSUM62	7	FWYH-MILV-CATS-P-G-NQDE-RK	W-M-T-P-G-D-R
BLOSUM62	8	FWYH-MILV-CA-NTS-P-G-DE-QRK	W-I-C-S-P-G-D-K
BLOSUM62	9	FWYH-ML-IV-CA-NTS-P-G-DE-QRK	H-L-I-C-S-P-G-E-K
BLOSUM62	10	FWY-ML-IV-CA-TS-NH-P-G-DE-QRK	W-L-V-C-T-N-P-G-E-R
BLOSUM62	11	FWY-ML-IV-CA-TS-NH-P-G-D-QE-RK	W-M-I-C-T-H-P-G-D-E-R
BLOSUM62	12	FWY-ML-IV-C-A-TS-NH-P-G-D-QE-RK	Y-M-V-C-A-T-N-P-G-D-E-R

BLOSUM62	13	FWY-ML-IV-C-A-T-S-NH-P-G-D-QE-RK	F-L-V-C-A-T-S-H-P-G-D-Q-R
BLOSUM62	15	FWY-ML-IV-C-A-T-S-N-H-P-G-D-QE-R-K	Y-L-I-C-A-T-S-N-H-P-G-D-Q-R-K
BLOSUM62	16	W-FY-ML-IV-C-A-T-S-N-H-P-G-D-QE-R-K	W-Y-M-I-C-A-T-S-N-H-P-G-D-E-R-K
BLOSUM62	17	W-FY-ML-IV-C-A-T-S-N-H-P-G-D-Q-E-R-K	W-Y-L-I-C-A-T-S-N-H-P-G-D-Q-E-R-K
BLOSUM62	18	W-FY-M-L-IV-C-A-T-S-N-H-P-G-D-Q-E-R-K	W-F-M-L-V-C-A-T-S-N-H-P-G-D-Q-E-R-K
BLOSUM62	19	W-F-Y-M-L-IV-C-A-T-S-N-H-P-G-D-Q-E-R-K	W-F-Y-M-L-V-C-A-T-S-N-H-P-G-D-Q-E-R-K
BLOSUM62 & MC	5	CFYW-MLIV-G-PATS-NHQEDRK	C-L-G-A-K
BLOSUM62 & MC	6	CFYW-MLIV-G-P-ATS-NHQEDRK	Y-V-G-P-T-D
BLOSUM62 & MC	7	CFYW-MLIV-G-P-ATS-NHQED-RK	C-V-G-P-A-E-K
BLOSUM62 & MC	8	CFYW-MLIV-G-P-ATS-NH-QED-RK	Y-V-G-P-S-N-E-R
BLOSUM62 & MC	9	CFYW-ML-IV-G-P-ATS-NH-QED-RK	F-M-V-G-P-S-H-E-R
BLOSUM62 & MC	10	C-FYW-ML-IV-G-P-ATS-NH-QED-RK	C-W-M-I-G-P-T-H-E-R
BLOSUM62 & MC	11	C-FYW-ML-IV-G-P-A-TS-NH-QED-RK	C-Y-L-V-G-P-A-T-N-E-R
BLOSUM62 & MC	12	C-FYW-ML-IV-G-P-A-TS-NH-QE-D-RK	C-F-M-V-G-P-A-S-N-Q-D-K
BLOSUM62 & MC	13	C-FYW-ML-IV-G-P-A-T-S-NH-QE-D-RK	C-Y-L-V-G-P-A-T-S-H-Q-D-K
BLOSUM62 & MC	14	C-FYW-ML-IV-G-P-A-T-S-N-H-QE-D-RK	C-F-M-V-G-P-A-T-S-N-H-Q-D-K
BLOSUM62 & MC	15	C-FYW-ML-IV-G-P-A-T-S-N-H-QE-D-R-K	C-W-L-V-G-P-A-T-S-N-H-Q-D-R-K
BLOSUM62 & MC	16	C-FY-W-ML-IV-G-P-A-T-S-N-H-QE-D-R-K	C-Y-W-M-I-G-P-A-T-S-N-H-E-D-R-K
BLOSUM62 & MC	17	C-FY-W-ML-IV-G-P-A-T-S-N-H-Q-E-D-R-K	C-F-W-M-I-G-P-A-T-S-N-H-Q-E-D-R-K
BLOSUM62 & MC	18	C-FY-W-M-L-IV-G-P-A-T-S-N-H-Q-E-D-R-K	C-Y-W-M-L-V-G-P-A-T-S-N-H-Q-E-D-R-K
BLOSUM62 & MC	19	C-F-Y-W-M-L-IV-G-P-A-T-S-N-H-Q-E-D-R-K	C-F-Y-W-M-L-V-G-P-A-T-S-N-H-Q-E-D-R-K
Boltzmann relation	5	VILMFWYA-C-ED-RK-GPSTHQN	M-C-E-R-G
Boltzmann relation	6	VILMFWY-A-C-ED-RK-GPSTHQN	I-A-C-E-R-H
Boltzmann relation	7	VILMFWY-A-C-ED-RK-G-PSTHQN	W-A-C-D-K-G-P
Boltzmann relation	8	VILMF-WY-A-C-ED-RK-G-PSTHQN	L-W-A-C-E-K-G-S
Boltzmann relation	9	VILMF-WY-A-C-ED-RK-G-P-STHQN	F-W-A-C-D-R-G-P-H
Boltzmann relation	10	VILMF-WY-A-C-ED-RK-G-P-ST-HQN	L-Y-A-C-D-K-G-P-T-H
Boltzmann relation	14	VILM-F-W-Y-A-C-ED-R-K-G-P-ST-H-QN	V-F-W-Y-A-C-E-R-K-G-P-S-H-N
Chemistry properties	8	DE-KRH-QN-ST-P-CM-WYF-GALIV	E-R-N-S-P-C-Y-A
Chemistry properties	10	DE-N-KRH-Q-T-SGAC-P-YWF-M-LIV	E-N-R-Q-T-G-P-Y-M-V
Chemistry space	5	NQHRK-YFW-MC-STDEGAPVL-I	N-W-M-L-I
Clustering analysis	5	APST-CGHIL-DENQ-FMWY-KRV	A-G-E-F-V
Clustering analysis	6	APST-CILMV-DENQ-FWY-G-HKR	S-L-Q-W-G-K
Contact potential	5	DE-KR-NQSTGPHY-ACW-FMLIV	D-R-G-C-F
Contact potential	7	DENQ-KR-G-P-AV-STHWY-CFMLI	N-K-G-P-V-Y-L
Distance matrix	5	LMWFY-CIV-NPHST-AG-DEQRK	M-V-N-G-D
Distance matrix	7	ND-HST-GPC-WFY-IVLM-RK-AQE	N-S-G-W-L-K-Q
Distance matrix	8	LM-WFY-CIV-NP-HST-AG-DE-QRK	L-Y-I-N-S-A-E-K
Distance matrix	12	LM-W-FY-C-IV-NP-H-ST-AG-DE-Q-RK	L-W-Y-C-V-P-H-T-A-E-Q-R
Dynamic Programming	10	DE-KRH-NQ-ST-ILV-FWY-C-M-AG-P	E-H-Q-S-L-Y-C-M-A-P
Dynamic clustering	5	TVMLFW-C-YA-G-RNDQEHKPST	M-C-A-G-T
Dynamic clustering	6	TVMLF-WY-C-AH-G-RNDQEKPST	M-Y-C-H-G-Q
Dynamic clustering	7	TVMLF-WY-C-AH-GP-R-NDQEKST	M-W-C-H-P-R-K

Dynamic clustering	8	TVMLF-WY-C-A-G-Q-R-NHDEKPST	F-W-C-A-G-Q-R-T
Dynamic clustering	9	TVMLF-WY-C-A-G-P-H-K-RQNDEST	L-W-C-A-G-P-H-K-N
Dynamic clustering	10	TVML-F-W-Y-C-A-H-G-RN-QPKDEST	V-F-W-Y-C-A-H-G-R-S
Dynamic clustering	11	TVMLF-W-Y-C-A-H-G-R-N-Q-PKDEST	L-W-Y-C-A-H-G-R-N-Q-S
Dynamic clustering	12	TVML-F-W-Y-C-A-H-G-N-Q-T-RDEKPS	L-F-W-Y-C-A-H-G-N-Q-T-S
Dynamic clustering	13	TVML-F-W-Y-C-A-H-G-R-N-Q-P-DEKST	L-F-W-Y-C-A-H-G-R-N-Q-P-D
Dynamic clustering	14	TVML-F-W-Y-C-A-H-G-R-N-Q-P-K-DEST	V-F-W-Y-C-A-H-G-R-N-Q-P-K-T
Dynamic clustering	15	TVML-F-W-Y-C-A-H-G-R-N-Q-P-K-D-EST	T-F-W-Y-C-A-H-G-R-N-Q-P-K-D-T
Dynamic clustering	16	TVML-F-W-Y-C-A-H-G-R-N-Q-P-K-S-T-DE	T-F-W-Y-C-A-H-G-R-N-Q-P-K-S-T-D
Dynamic clustering	17	TVL-M-F-W-Y-C-A-H-G-R-N-Q-P-K-S-T-DE	V-M-F-W-Y-C-A-H-G-R-N-Q-P-K-S-T-D
Dynamic clustering	18	TVL-M-F-W-Y-C-A-H-G-R-N-Q-P-K-S-T-D-E	T-M-F-W-Y-C-A-H-G-R-N-Q-P-K-S-T-D-E
ECGA	5	ACIY-MFLV-GHTN-SWDE-PRKQ	I-M-T-W-P
Fuzzy clustering	5	EHKQDNILPTACGMS-V-FY-R-W	Q-V-Y-R-W
Fuzzy clustering	6	DEHIKLMNPQRST-FWY-A-C-G-V	M-F-A-C-G-V
Fuzzy clustering	7	ADEGHIKLNPQST-C-M-R-V-FY-W	H-C-M-R-V-Y-W
Fuzzy clustering	8	ADEGNPQST-HIKL-R-V-FY-C-M-W	S-K-R-V-F-C-M-W
Fuzzy clustering	10	DEHIKLNPQT-FY-M-R-S-W-A-C-G-V	L-Y-M-R-S-W-A-C-G-V
Fuzzy clustering	11	DENQT-HIKLR-FY-M-P-S-W-A-C-G-V	Q-K-F-M-P-S-W-A-C-G-V
Fuzzy clustering	13	EHKQ-DN-IL-PT-FY-M-R-S-W-A-C-G-V	Q-D-I-T-Y-M-R-S-W-A-C-G-V
Fuzzy clustering	14	EHKQ-DN-IL-PT-A-C-G-M-S-V-F-Y-R-W	H-N-L-T-A-C-G-M-S-V-F-Y-R-W
Fuzzy clustering	15	EQ-D-N-T-HIKL-R-FY-M-P-S-W-A-C-G-V	Q-D-N-T-L-R-Y-M-P-S-W-A-C-G-V
Fuzzy clustering	16	EQ-H-K-DN-IL-P-T-FY-M-R-S-W-A-C-G-V	Q-H-K-N-I-P-T-F-M-R-S-W-A-C-G-V
Fuzzy clustering	17	EQ-H-K-DN-IL-P-T-M-R-S-F-Y-W-A-C-G-V	Q-H-K-D-L-P-T-M-R-S-F-Y-W-A-C-G-V
Fuzzy clustering	18	E-Q-D-N-T-IL-H-K-R-FY-M-P-S-W-A-C-G-V	E-Q-D-N-T-I-H-K-R-Y-M-P-S-W-A-C-G-V
Fuzzy clustering	19	E-H-K-Q-D-N-IL-P-T-A-C-G-M-S-V-F-Y-R-W	E-H-K-Q-D-N-L-P-T-A-C-G-M-S-V-F-Y-R-W
GDM	5	AG-CDQ-EHILNVYFMRSW-K-PT	G-D-H-K-P
GDM	7	AG-CDQ-EHNY-FMRSW-ILV-K-PT	A-D-N-S-V-K-P
GDM	8	AG-C-DQ-EHNY-FMRSW-ILV-K-PT	A-C-D-E-M-V-K-P
GDM	9	AG-C-DQ-EHNY-FMW-ILV-K-PT-RS	A-C-D-Y-W-V-K-T-R
GDM	10	A-C-DQ-EHNY-FMW-G-ILV-K-PT-RS	A-C-D-H-F-G-I-K-P-R
GDM	11	A-C-DQ-EHNY-FM-G-ILV-K-PT-RS-W	A-C-Q-H-M-G-V-K-P-S-W
GDM	12	A-C-DQ-EHNY-FM-G-IL-K-PT-RS-V-W	A-C-Q-N-F-G-L-K-P-R-V-W
GDM	13	A-C-DQ-E-FM-G-HNY-IL-K-PT-RS-V-W	A-C-Q-E-M-G-Y-I-K-P-S-V-W
GDM	14	A-C-D-E-FM-G-HNY-IL-K-PT-Q-RS-V-W	A-C-D-E-M-G-H-I-K-T-Q-S-V-W
GDM	15	A-C-D-E-FM-G-HNY-IL-K-PT-Q-R-S-V-W	A-C-D-E-M-G-N-I-K-T-Q-R-S-V-W
GDM	16	A-C-D-E-F-G-HNY-IL-K-M-PT-Q-R-S-V-W	A-C-D-E-F-G-Y-L-K-M-P-Q-R-S-V-W
GDM	17	A-C-D-E-F-G-HNY-IL-K-M-P-Q-R-S-T-V-W	A-C-D-E-F-G-Y-L-K-M-P-Q-R-S-T-V-W
GDM	18	A-C-D-E-F-G-HNY-I-K-L-M-P-Q-R-S-T-V-W	A-C-D-E-F-G-H-I-K-L-M-P-Q-R-S-T-V-W
GDM	19	A-C-D-E-F-G-HN-I-K-L-M-P-Q-R-S-T-V-W-Y	A-C-D-E-F-G-N-I-K-L-M-P-Q-R-S-T-V-W-Y
GONNET	5	W-C-GPHNDERQKAST-FY-VMIL	W-C-A-F-L
GONNET	6	W-C-G-PHNDERQKAST-FY-VMIL	W-C-G-E-F-I
GONNET	7	W-C-G-P-HNDERQKAST-FY-VMIL	W-C-G-P-D-F-L
GONNET	8	W-C-G-P-H-NDERQKAST-FY-VMIL	W-C-G-P-H-N-Y-L

GONNET	9	W-C-G-P-H-NDERQK-AST-FY-VMIL	W-C-G-P-H-N-T-F-L
GONNET	10	W-C-G-P-H-NDERQK-AST-F-Y-VMIL	W-C-G-P-H-E-A-F-Y-L
GONNET	11	W-C-G-P-H-NDE-RQK-AST-F-Y-VMIL	W-C-G-P-H-D-Q-S-F-Y-I
GONNET	12	W-C-G-P-H-N-DE-RQK-AST-F-Y-VMIL	W-C-G-P-H-N-E-Q-S-F-Y-L
GONNET	13	W-C-G-P-H-N-DE-RQK-AST-F-Y-V-MIL	W-C-G-P-H-N-E-K-A-F-Y-V-M
GONNET	14	W-C-G-P-H-N-D-E-RQK-AST-F-Y-V-MIL	W-C-G-P-H-N-D-E-K-A-F-Y-V-L
GONNET	15	W-C-G-P-H-N-D-E-R-QK-AST-F-Y-V-MIL	W-C-G-P-H-N-D-E-R-K-T-F-Y-V-L
GONNET	17	W-C-G-P-H-N-D-E-R-QK-A-ST-F-Y-V-M-IL	W-C-G-P-H-N-D-E-R-K-A-T-F-Y-V-M-L
Genetic algorithm	5	AG-C-DEKNPQRST-FILMVWY-H	G-C-P-Y-H
Hierarchical clustering	5	PWDHI-GYCS-REL-NF-TVKMAQ	I-S-L-F-A
Hierarchical clustering	6	STAND-G-RQEK-HP-IVLMWYF-C	N-G-E-H-Y-C
Hierarchical clustering	7	C-DE-RK-STNQ-HP-VILMF-WYAG	C-D-R-Q-H-M-G
Hierarchical clustering	8	G-IVWFY-ALM-EQRK-P-ND-C-HST	G-F-L-E-P-N-C-S
Hierarchical clustering	9	PW-DHI-GY-CS-REL-NF-TV-KM-AQ	P-H-G-S-E-F-V-M-Q
Hierarchical clustering	10	ST-A-ND-G-RQ-EK-HP-IVLM-WYF-C	T-A-D-G-Q-E-H-I-Y-C
Hierarchical clustering	11	RK-QE-ND-H-ST-P-A-G-IVLM-FYW-C	K-Q-D-H-T-P-A-G-V-F-C
Hierarchical clustering	12	ST-A-N-D-G-RQ-EK-H-P-IVLM-WYF-C	S-A-N-D-G-R-E-H-P-M-F-C
Hierarchical clustering	13	RK-QE-ND-H-S-T-P-A-G-IV-LM-FYW-C	R-E-D-H-S-T-P-A-G-V-M-Y-C
Hierarchical clustering	14	RK-QE-N-D-H-S-T-P-A-G-IV-LM-FYW-C	K-E-N-D-H-S-T-P-A-G-I-L-F-C
Hierarchical clustering	15	C-DE-R-K-ST-NQ-H-P-VIL-M-F-W-Y-A-G	C-E-R-K-S-Q-H-P-L-M-F-W-Y-A-G
Hierarchical clustering	16	C-DE-R-K-ST-NQ-H-P-V-IL-M-F-W-Y-A-G	C-D-R-K-S-Q-H-P-V-L-M-F-W-Y-A-G
Hierarchical clustering	17	RK-Q-E-N-D-H-S-T-P-A-G-IV-L-M-FY-W-C	K-Q-E-N-D-H-S-T-P-A-G-V-L-M-Y-W-C
Hierarchical clustering	18	S-T-A-N-D-G-R-Q-E-K-H-P-IV-L-M-WY-F-C	S-T-A-N-D-G-R-Q-E-K-H-P-I-L-M-Y-F-C
Hierarchical clustering	19	S-T-A-N-D-G-R-Q-E-K-H-P-I-V-L-M-WY-F-C	S-T-A-N-D-G-R-Q-E-K-H-P-I-V-L-M-W-F-C
Information theory	8	DKR-EA-GP-STNQ-H-C-WY-FMLIV	R-A-G-T-H-C-Y-L
JTT	5	AGPST-CFWY-DEN-HKQR-ILMV	G-Y-N-K-M
JTT	6	APST-CW-DEGN-FHY-ILMV-KQR	S-C-N-F-M-K
JTT	7	AGST-CW-DEN-FY-HP-ILMV-KQR	G-C-D-F-H-M-K
JTT	8	AST-CG-DEN-FY-HP-ILV-KQR-MW	S-C-E-F-H-V-R-M
JTT	9	AST-CW-DE-FY-GN-HQ-ILV-KR-MP	T-C-D-Y-G-Q-I-R-M
JTT	10	AST-CW-DE-FY-GN-HQ-IV-KR-LM-P	T-W-D-Y-N-Q-V-K-M-P
JTT	11	AST-C-DE-FY-GN-HQ-IV-KR-LM-P-W	A-C-D-F-N-H-I-R-L-P-W
JTT	12	AST-C-DE-FY-G-HQ-IV-KR-LM-N-P-W	A-C-D-Y-G-H-V-K-L-N-P-W
JTT	13	AST-C-DE-FY-G-H-IV-KR-LM-N-P-Q-W	S-C-E-Y-G-H-V-R-L-N-P-Q-W
JTT	14	AST-C-DE-FL-G-H-IV-KR-M-N-P-Q-W-Y	T-C-E-F-G-H-V-R-M-N-P-Q-W-Y
JTT	15	AST-C-DE-F-G-H-IV-KR-L-M-N-P-Q-W-Y	T-C-D-F-G-H-I-K-L-M-N-P-Q-W-Y
JTT	16	AT-C-DE-F-G-H-IV-KR-L-M-N-P-Q-S-W-Y	A-C-D-F-G-H-I-R-L-M-N-P-Q-S-W-Y
JTT	17	AT-C-DE-F-G-H-IV-K-L-M-N-P-Q-R-S-W-Y	A-C-E-F-G-H-V-K-L-M-N-P-Q-R-S-W-Y
JTT	18	A-C-DE-F-G-H-IV-K-L-M-N-P-Q-R-S-T-W-Y	A-C-E-F-G-H-I-K-L-M-N-P-Q-R-S-T-W-Y
JTT	19	A-C-D-E-F-G-H-IV-K-L-M-N-P-Q-R-S-T-W-Y	A-C-D-E-F-G-H-I-K-L-M-N-P-Q-R-S-T-W-Y
K-means	8	ED-QST-NH-YPRK-LMI-VAF-GW-C	D-T-H-R-M-V-W-C
MIG	5	G-DN-AEHKQRST-CFILMVWY-P	G-D-T-C-P
MIG	6	G-DN-AEFHILMKQRVWY-CT-S-P	G-N-H-T-S-P

MIG	7	G-DN-AEFILMKQRVWY-CH-T-S-P	G-N-E-C-T-S-P
MIG	8	G-D-N-AEFILMKQRVWY-CH-T-S-P	G-D-N-W-C-T-S-P
MIG	9	G-D-N-AEFILMKQRVWY-C-H-T-S-P	G-D-N-Q-C-H-T-S-P
MIG	10	G-D-N-AEFILMKQRVW-Y-C-H-T-S-P	G-D-N-F-Y-C-H-T-S-P
MIG	11	G-D-N-AEFILMKQRV-W-Y-C-H-T-S-P	G-D-N-V-W-Y-C-H-T-S-P
MIG	12	G-D-N-AEFILMKQV-R-W-Y-C-H-T-S-P	G-D-N-E-R-W-Y-C-H-T-S-P
MIG	13	G-D-N-AEFILMKV-Q-R-W-Y-C-H-T-S-P	G-D-N-K-Q-R-W-Y-C-H-T-S-P
MIG	14	G-D-N-AEFILKV-M-Q-R-W-Y-C-H-T-S-P	G-D-N-I-M-Q-R-W-Y-C-H-T-S-P
MJ	5	MFILV-AWC-YQHPGTSN-RK-DE	V-A-H-R-E
MJ	6	MFILV-A-C-WYQHPGTSN-RK-DE	I-A-C-G-K-E
MJ	7	MFILV-A-C-WYQHP-GTSN-RK-DE	F-A-C-Y-N-R-E
MJ	8	LF-I-MVW-CY-HAT-GP-RQS-NEDK	L-I-V-Y-H-P-S-D
MJ	9	MF-ILV-A-C-WYQHP-G-TSN-RK-DE	F-V-A-C-P-G-S-R-D
MJ	10	QSNTGA-P-ED-LIVM-FW-Y-H-R-K-C	G-P-D-I-W-Y-H-R-K-C
MJ	11	MF-IL-V-A-C-WYQHP-G-TSN-RK-D-E	F-I-V-A-C-W-G-N-R-D-E
MJ	12	MF-IL-V-A-C-WYQHP-G-TS-N-RK-D-E	F-L-V-A-C-P-G-T-N-K-D-E
MJ	13	QSNT-G-A-P-E-D-LIVM-FW-Y-H-R-K-C	N-G-A-P-E-D-L-W-Y-H-R-K-C
MJ	14	QSNT-G-A-P-E-D-LIVM-F-W-Y-H-R-K-C	N-G-A-P-E-D-M-F-W-Y-H-R-K-C
MJ	15	QSN-T-G-A-P-E-D-LIVM-F-W-Y-H-R-K-C	Q-T-G-A-P-E-D-M-F-W-Y-H-R-K-C
MJ	16	MF-I-L-V-A-C-WYQ-H-P-G-T-S-N-RK-D-E	F-I-L-V-A-C-Q-H-P-G-T-S-N-R-D-E
MJ	18	Q-S-N-T-G-A-P-E-D-LIV-F-M-W-Y-H-R-K-C	Q-S-N-T-G-A-P-E-D-V-F-M-W-Y-H-R-K-C
MJ	19	Q-S-N-T-G-A-P-E-D-LI-V-F-M-W-Y-H-R-K-C	Q-S-N-T-G-A-P-E-D-I-V-F-M-W-Y-H-R-K-C
MJ & BLOSUM62	13	MF-IL-V-A-C-WYQHP-G-T-S-N-RK-D-E	F-I-V-A-C-P-G-T-S-N-K-D-E
MJ & BLOSUM62	14	IMV-L-F-WY-G-P-C-A-S-T-N-HRKQ-E-D	V-L-F-W-G-P-C-A-S-T-N-K-E-D
MJ & BLOSUM62	19	P-G-E-K-R-Q-D-S-N-T-H-C-I-V-W-YF-A-L-M	P-G-E-K-R-Q-D-S-N-T-H-C-I-V-W-Y-A-L-M
MMI	5	FWY-CILMV-DEGKNS-APQT-HR	Y-I-D-A-R
MMI	6	FWY-CILMV-DE-GKNQS-APT-HR	F-L-D-S-T-R
MMI	7	FWY-CILMV-DE-K-GNPQS-AT-HR	Y-V-D-K-G-A-H
MMI	8	FWY-ILMV-C-DE-K-GNPQS-AT-HR	W-L-C-D-K-P-T-R
MMI	9	FWY-ILMV-C-DE-K-GNQS-PT-A-HR	Y-M-C-E-K-S-T-A-H
MMI	10	WY-F-ILMV-C-DE-K-GNQS-PT-A-HR	W-F-V-C-D-K-N-T-A-R
MMI	11	WY-F-ILMV-C-DE-K-G-PNQS-T-A-HR	W-F-L-C-E-K-G-Q-T-A-R
MMI	12	WY-F-IL-MV-C-DE-K-G-PNQS-T-A-HR	Y-F-I-M-C-D-K-G-S-T-A-R
MMI	13	WY-F-IL-MV-C-DE-K-G-P-NQS-T-A-HR	Y-F-I-M-C-D-K-G-P-Q-T-A-H
MMI	14	W-Y-F-IL-MV-C-DE-K-G-P-NQS-T-A-HR	W-Y-F-L-V-C-E-K-G-P-S-T-A-H
MMI	15	W-Y-F-IL-MV-C-DE-K-G-P-NQS-T-A-H-R	W-Y-F-I-M-C-E-K-G-P-S-T-A-H-R
MMI	16	W-Y-F-IL-M-V-C-DE-K-G-P-NQS-T-A-H-R	W-Y-F-I-M-V-C-D-K-G-P-S-T-A-H-R
MMI	17	W-Y-F-I-L-M-V-C-DE-K-G-P-NQS-T-A-H-R	W-Y-F-I-L-M-V-C-D-K-G-P-N-T-A-H-R
MMI	18	W-Y-F-I-L-M-V-C-DE-K-G-P-N-QS-T-A-H-R	W-Y-F-I-L-M-V-C-D-K-G-P-N-S-T-A-H-R
MMI	19	W-Y-F-I-L-M-V-C-D-E-K-G-P-N-QS-T-A-H-R	W-Y-F-I-L-M-V-C-D-E-K-G-P-N-Q-T-A-H-R
PAM	5	AGSPDEQNHTKR-MIVL-FY-C-W	H-V-Y-C-W
PAM	6	AGSP-DEQNHTKR-MIL-FY-CV-W	S-K-L-Y-V-W
PAM	7	AGP-DEQNH-TKRIMV-L-FY-CS-W	G-D-M-L-F-C-W

PAM	8	AG-DEQN-TKRIMV-L-HY-CS-FP-W	A-E-V-L-H-S-P-W
PAM	9	AG-P-DEQN-TKRIM-L-F-HY-VCS-W	G-P-E-K-L-F-H-S-W
PAM	10	AG-P-DEQN-TKRM-L-F-HY-VCS-I-W	G-P-Q-K-L-F-H-C-I-W
PAM	11	AG-P-DEQN-TK-RI-ML-F-H-Y-VCS-W	G-P-Q-T-R-L-F-H-Y-S-W
PAM	12	FAS-P-G-DEQ-NL-TK-R-H-W-Y-IM-VC	F-P-G-D-L-T-R-H-W-Y-M-V
PAM	13	FAS-P-G-DEQ-NL-T-K-R-H-W-Y-IM-VC	S-P-G-E-N-T-K-R-H-W-Y-M-V
PAM	14	FA-P-G-T-DE-QM-NL-K-R-H-W-Y-IV-SC	F-P-G-T-D-M-N-K-R-H-W-Y-I-S
PAM	15	FAS-P-G-DE-T-Q-NL-K-R-H-W-Y-I-M-VC	S-P-G-D-T-Q-N-K-R-H-W-Y-I-M-C
PAM	16	FA-P-G-ST-DE-Q-N-K-R-H-W-Y-M-L-I-VC	A-P-G-S-E-Q-N-K-R-H-W-Y-M-L-I-V
PAM	17	FA-P-G-ST-DE-Q-N-K-R-H-W-Y-M-L-I-V-C	F-P-G-T-D-Q-N-K-R-H-W-Y-M-L-I-V-C
PAM	18	FA-P-G-S-T-DE-Q-N-K-R-H-W-Y-M-L-I-V-C	F-P-G-S-T-D-Q-N-K-R-H-W-Y-M-L-I-V-C
PAM	19	FA-P-G-S-T-D-E-Q-N-K-R-H-W-Y-M-L-I-V-C	F-P-G-S-T-D-E-Q-N-K-R-H-W-Y-M-L-I-V-C
PSO	5	EL-DTV-RGKFW-AQHIPY-NCMS	L-T-R-P-N
PSO	6	CILKMSV-Y-FW-G-R-ANDQEHPT	L-Y-F-G-R-T
PSO	8	NQEHKPS-L-WY-R-I-G-V-ADCMFT	S-L-W-R-I-G-V-C
Physical-chemical	6	RDENQKH-LIVAMF-STYW-P-G-C	E-I-Y-P-G-C
Physical-chemical	7	AV-CGNP-D-EKRQ-FWYH-ILM-ST	V-P-D-E-F-M-T
Physical-chemical	9	AV-CGNP-D-EKR-Q-FWY-H-ILM-ST	A-C-D-K-Q-Y-H-L-T
Physical-chemical	11	AV-C-GNP-D-EKR-Q-FWY-H-IL-M-ST	A-C-G-D-E-Q-F-H-I-M-S
Physical-chemical	19	G-I-LV-F-Y-W-A-M-E-Q-R-K-P-N-D-H-S-T-C	G-I-V-F-Y-W-A-M-E-Q-R-K-P-N-D-H-S-T-C
Protein blocks	5	G-IVFYW-ALMEQRK-P-NDHSTC	G-V-M-P-N
Protein blocks	8	DKR-EA-GP-STNQ-H-C-WY-FMLIV	R-E-G-T-H-C-Y-L
Protein blocks	9	G-IV-FYW-ALM-EQRK-P-ND-HS-TC	G-I-F-M-E-P-D-S-T
Protein blocks	10	DNS-EKRQ-TH-GP-AM-C-W-F-YL-IV	N-E-T-G-A-C-W-F-L-I
Protein blocks	11	G-IV-FYW-A-LM-EQRK-P-ND-HS-T-C	G-I-F-A-L-R-P-N-S-T-C
Protein blocks	13	G-IV-FYW-A-L-M-E-QRK-P-ND-HS-T-C	G-V-W-A-L-M-E-K-P-N-H-T-C
SDM	5	P-HGAREKQSTND-W-C-MVILFY	P-H-W-C-F
SDM	6	P-H-GAREKQSTND-W-C-MVILFY	P-H-E-W-C-I
SDM	7	P-H-G-AREKQSTND-W-C-MVILFY	P-H-G-D-W-C-V
SDM	8	P-H-G-AREKQST-ND-W-C-MVILFY	P-H-G-E-D-W-C-F
SDM	9	P-H-G-AREKQST-ND-W-C-MVIL-FY	P-H-G-A-N-W-C-V-F
SDM	10	P-H-G-A-REKQST-ND-W-C-MVIL-FY	P-H-G-A-S-D-W-C-M-F
SDM	11	P-H-G-A-REK-QST-ND-W-C-MVIL-FY	P-H-G-A-R-T-D-W-C-I-F
SDM	12	P-H-G-A-REK-QST-N-D-W-C-MVIL-FY	P-H-G-A-E-Q-N-D-W-C-I-F
SDM	13	P-H-G-A-REK-QST-N-D-W-C-MVIL-F-Y	P-H-G-A-K-Q-N-D-W-C-I-F-Y
SDM	14	P-H-G-A-REK-QST-N-D-W-C-M-VIL-F-Y	P-H-G-A-R-T-N-D-W-C-M-V-F-Y
SDM	15	P-H-G-A-R-EK-QST-N-D-W-C-M-VIL-F-Y	P-H-G-A-R-K-Q-N-D-W-C-M-V-F-Y
SDM	16	P-H-G-A-R-EK-Q-ST-N-P-W-C-M-VIL-F-Y	P-H-G-A-R-E-Q-S-N-P-W-C-M-V-F-Y
SDM	17	P-H-G-A-R-EK-Q-S-T-N-P-W-C-M-VIL-F-Y	P-H-G-A-R-E-Q-S-T-N-P-W-C-M-I-F-Y
Sequence alignments	5	DSHFM-ERQL-KPAC-NTWY-GIV	H-R-K-T-G
Sequence alignments	6	DENQ-KRH-STGPA-C-WYF-MLIV	N-R-T-C-W-M
Sequence alignments	10	DN-EQ-KR-STA-G-P-HW-C-YF-MLIV	N-Q-K-S-G-P-W-C-Y-L
Sequence alignments	13	D-E-KQR-NS-T-G-P-H-A-C-WYF-ML-IV	D-E-Q-N-T-G-P-H-A-C-Y-M-V

Sequence alignments	16	D-E-N-KR-Q-ST-G-P-H-A-C-W-Y-F-ML-IV	D-E-N-K-Q-T-G-P-H-A-C-W-Y-F-M-V
Structure	5	PG-EKRQ-DSNTHC-IVWYF-ALM	P-K-S-I-A
Structure	6	PG-EKRQ-DSN-THC-IVWYF-ALM	P-Q-N-C-Y-L
Structure	7	PG-EKRQ-DSN-THC-IVWYF-A-LM	P-Q-D-C-I-A-M
Structure	8	P-G-EKRQ-DSN-THC-IVWYF-A-LM	P-G-R-N-C-V-A-M
Structure	9	P-G-EKRQ-DSN-THC-IV-WYF-A-LM	P-G-K-N-C-I-W-A-M
Structure	10	P-G-EKRQ-DSN-T-HC-IV-WYF-A-LM	P-G-R-D-T-H-I-W-A-L
Structure	11	P-G-EKRQ-D-SN-T-HC-IV-WYF-A-LM	P-G-Q-D-N-T-C-I-F-A-M
Structure	12	P-G-EKRQ-D-SN-T-H-C-IV-WYF-A-LM	P-G-K-D-N-T-H-C-I-W-A-L
Structure	13	P-G-E-KRQ-D-SN-T-H-C-IV-WYF-A-LM	P-G-E-K-D-N-T-H-C-I-F-A-L
Structure	15	P-G-E-KRQ-D-S-N-T-H-C-IV-W-YF-A-LM	P-G-E-R-D-S-N-T-H-C-V-W-F-A-M
Structure	16	P-G-E-KRQ-D-S-N-T-H-C-IV-W-YF-A-L-M	P-G-E-Q-D-S-N-T-H-C-I-W-F-A-L-M
Structure	17	P-G-E-K-RQ-D-S-N-T-H-C-IV-W-YF-A-L-M	P-G-E-K-R-D-S-N-T-H-C-V-W-F-A-L-M
Structure	18	P-G-E-K-RQ-D-S-N-T-H-C-I-V-W-YF-A-L-M	P-G-E-K-R-D-S-N-T-H-C-I-V-W-Y-A-L-M
Structure	19	P-G-E-K-R-Q-D-S-N-T-H-C-I-V-W-YF-A-L-M	P-G-E-K-R-Q-D-S-N-T-H-C-I-V-W-F-A-L-M
Structure alignments	5	DE-NKRQS-THA-GP-CWYFMLIV	E-R-H-G-V
Structure alignments	6	DE-KR-NQST-GP-HWYF-ACMLIV	D-R-N-G-H-M
Structure alignments	12	D-N-EKR-QST-G-P-H-A-C-W-YF-MLIV	D-N-E-S-G-P-H-A-C-W-F-L
Structure alignments	15	D-N-E-KRQ-S-T-G-P-H-A-C-W-YF-ML-IV	D-N-E-R-S-T-G-P-H-A-C-W-Y-M-I
Structure alignments	17	D-EK-N-R-Q-S-T-G-P-H-A-C-W-Y-F-M-LIV	D-K-N-R-Q-S-T-G-P-H-A-C-W-Y-F-M-L
UPGMA	5	ASTNDRQEKIVLMGP-FY-H-C-W	S-Y-H-C-W
UPGMA	6	ASTNDRQEKIVLMG-P-FY-H-C-W	S-P-F-H-C-W
UPGMA	7	ASTNDRQEKIVLM-G-P-FY-H-C-W	N-G-P-F-H-C-W
UPGMA	8	ASTNDRQEK-IVLM-G-P-FY-H-C-W	E-V-G-P-Y-H-C-W
UPGMA	10	ASTND-RQEK-IVLM-G-P-F-Y-H-C-W	T-Q-I-G-P-F-Y-H-C-W
UPGMA	11	ASTN-D-RQEK-IVLM-G-P-F-Y-H-C-W	S-D-Q-V-G-P-F-Y-H-C-W
UPGMA	12	ASTN-D-RQ-EK-IVLM-G-P-F-Y-H-C-W	A-D-R-E-M-G-P-F-Y-H-C-W
UPGMA	14	AST-N-D-RQ-EK-IVL-M-G-P-F-Y-H-C-W	T-N-D-R-K-V-M-G-P-F-Y-H-C-W
UPGMA	15	AST-N-D-R-Q-EK-IVL-M-G-P-F-Y-H-C-W	S-N-D-R-Q-E-V-M-G-P-F-Y-H-C-W
UPGMA	16	AST-N-D-R-Q-E-K-IVL-M-G-P-F-Y-H-C-W	T-N-D-R-Q-E-K-L-M-G-P-F-Y-H-C-W
UPGMA	17	AST-N-D-R-Q-E-K-IV-L-M-G-P-F-Y-H-C-W	S-N-D-R-Q-E-K-I-L-M-G-P-F-Y-H-C-W
UPGMA	18	AS-T-N-D-R-Q-E-K-IV-L-M-G-P-F-Y-H-C-W	S-T-N-D-R-Q-E-K-I-L-M-G-P-F-Y-H-C-W
UVG	5	LIVGAP-QNMTSC-ED-KR-YFWH	A-Q-E-R-W
UVG	6	LIVGAP-QNMTSC-ED-KR-YFW-H	P-S-E-K-W-H
UVG	7	LIVGAP-QNM-TSC-ED-KR-YFW-H	A-N-T-E-K-W-H
UVG	8	LIV-GAP-QNM-TSC-ED-KR-YFW-H	V-P-M-T-E-R-Y-H
UVG	10	LIV-GA-P-QN-M-TSC-ED-KR-YFW-H	I-A-P-Q-M-S-D-K-W-H
UVG	13	LIV-GA-P-QN-M-TS-C-ED-KR-Y-F-W-H	I-A-P-Q-M-T-C-E-K-Y-F-W-H
UVG	14	LIV-GA-P-QN-M-TS-C-ED-K-R-Y-F-W-H	I-A-P-N-M-S-C-D-K-R-Y-F-W-H
UVG	15	LIV-GA-P-QN-M-T-S-C-ED-K-R-Y-F-W-H	I-A-P-N-M-T-S-C-E-K-R-Y-F-W-H
UVG	16	LIV-GA-P-Q-N-M-T-S-C-ED-K-R-Y-F-W-H	L-A-P-Q-N-M-T-S-C-D-K-R-Y-F-W-H
UVG	17	LIV-G-A-P-Q-N-M-T-S-C-ED-K-R-Y-F-W-H	I-G-A-P-Q-N-M-T-S-C-E-K-R-Y-F-W-H
UVG	18	LI-V-G-A-P-Q-N-M-T-S-C-ED-K-R-Y-F-W-H	I-V-G-A-P-Q-N-M-T-S-C-E-K-R-Y-F-W-H

UVG	19	LI-V-G-A-P-Q-N-M-T-S-C-E-D-K-R-Y-F-W-H	L-V-G-A-P-Q-N-M-T-S-C-E-D-K-R-Y-F-W-H
Unweighted pair group	5	AWMGST-HPY-CVIFL-DNQ-ERK	T-H-C-D-R
Unweighted pair group	6	AGTLV-DKNFQ-EIPRS-CMY-H-W	V-Q-E-M-H-W
Unweighted pair group	7	AEM-L-CDT-NY-FIKHQVRW-GS-P	M-L-D-Y-H-G-P
Unweighted pair group	8	AWM-GST-HPY-CVI-FL-DNQ-ER-K	A-T-Y-I-L-Q-R-K
Unweighted pair group	9	AWM-GS-T-HPY-CVI-FL-DNQ-ER-K	A-G-T-Y-C-L-N-R-K
Unweighted pair group	10	AEM-L-CDT-NY-FIKH-QV-RW-G-S-P	M-L-D-Y-K-Q-W-G-S-P
Unweighted pair group	11	A-GT-LV-DKN-FQ-E-IPR-S-CMY-H-W	A-T-L-D-F-E-R-S-C-H-W
Unweighted pair group	12	AWM-G-S-T-H-PY-CVI-FL-DNQ-E-R-K	M-G-S-T-H-Y-V-F-Q-E-R-K
Unweighted pair group	13	A-G-T-LV-DKN-FQ-E-IPR-S-CM-Y-H-W	A-G-T-L-N-Q-E-P-S-M-Y-H-W
Unweighted pair group	14	A-EM-L-C-DT-NY-FIK-H-Q-V-RW-G-S-P	A-E-L-C-D-Y-F-H-Q-V-W-G-S-P
Unweighted pair group	15	A-C-V-HP-L-D-Q-S-ER-GN-F-IMT-K-W-Y	A-C-V-H-L-D-Q-S-E-G-F-I-K-W-Y
Unweighted pair group	16	A-C-V-HP-L-D-Q-S-ER-GN-F-I-MT-K-W-Y	A-C-V-P-L-D-Q-S-E-N-F-I-M-K-W-Y
Unweighted pair group	17	A-C-V-H-P-L-D-Q-S-ER-GN-F-I-MT-K-W-Y	A-C-V-H-P-L-D-Q-S-R-G-F-I-T-K-W-Y
Unweighted pair group	18	A-G-T-L-V-DK-N-F-Q-E-I-P-R-S-CM-Y-H-W	A-G-T-L-V-D-N-F-Q-E-I-P-R-S-C-Y-H-W
Unweighted pair group	19	A-E-M-L-C-D-T-NY-F-I-K-H-Q-V-R-W-G-S-P	A-E-M-L-C-D-T-N-F-I-K-H-Q-V-R-W-G-S-P
WAG	5	HRKQNEDSTGPA-CV-IML-FY-W	E-V-M-Y-W
WAG	6	HRKQNEDSTPA-G-CV-IML-FY-W	H-G-V-I-Y-W
WAG	7	HRKQNEDSTA-G-P-CV-IML-FY-W	K-G-P-V-I-F-W
WAG	8	HRKQSTA-NED-G-P-CV-IML-FY-W	K-E-G-P-C-L-Y-W
WAG	9	HRKQ-NED-ASTG-P-C-IV-MLF-Y-W	K-E-G-P-C-V-M-Y-W
WAG	10	HRKSA-Q-NED-G-P-C-TIV-MLF-Y-W	H-Q-D-G-P-C-I-F-Y-W
WAG	11	RKQ-NG-ED-AST-P-C-IV-HML-F-Y-W	R-G-E-A-P-C-I-H-F-Y-W
WAG	12	RKQ-ED-NAST-G-P-C-IV-H-ML-F-Y-W	K-D-S-G-P-C-I-H-L-F-Y-W
WAG	13	RK-QE-D-NG-HA-ST-P-C-IV-ML-F-Y-W	K-Q-D-G-H-S-P-C-V-M-F-Y-W
WAG	14	R-K-QE-D-NG-HA-ST-P-C-IV-ML-F-Y-W	R-K-Q-D-G-H-S-P-C-V-L-F-Y-W
WAG	15	R-K-QE-D-NG-HA-ST-P-C-IV-M-L-F-Y-W	R-K-Q-D-G-H-T-P-C-I-M-L-F-Y-W
WAG	16	R-K-Q-E-D-NG-HA-ST-P-C-IV-M-L-F-Y-W	R-K-Q-E-D-G-H-T-P-C-I-M-L-F-Y-W
WAG	17	R-K-Q-E-D-NG-HA-S-T-P-C-IV-M-L-F-Y-W	R-K-Q-E-D-N-H-S-T-P-C-I-M-L-F-Y-W
WAG	18	R-K-Q-E-D-NG-HA-S-T-P-C-I-V-M-L-F-Y-W	R-K-Q-E-D-G-H-S-T-P-C-I-V-M-L-F-Y-W
WAG	19	R-K-Q-E-D-NG-H-A-S-T-P-C-I-V-M-L-F-Y-W	R-K-Q-E-D-G-H-A-S-T-P-C-I-V-M-L-F-Y-W
variance maximization	5	WFYH-MLIV-GP-CATS-NDEQRK	W-V-P-A-K
variance maximization	6	WFYH-MLIV-G-P-CATS-NDEQRK	W-L-G-P-A-R
variance maximization	7	WFY-MLIV-G-P-CATS-NDE-HQRK	W-V-G-P-S-E-H
variance maximization	8	WFY-MLIV-C-G-P-ATS-NDE-HQRK	W-L-C-G-P-S-D-H
variance maximization	9	WFY-MLIV-C-G-P-ATS-NDE-H-QRK	Y-L-C-G-P-T-E-H-K
variance maximization	10	W-FY-MLIV-C-G-P-ATS-NDE-H-QRK	W-F-M-C-G-P-T-E-H-R
variance maximization	11	W-FY-MLIV-C-G-P-ATS-N-DE-H-QRK	W-F-I-C-G-P-T-N-D-H-Q
variance maximization	12	W-FY-MLIV-A-C-G-P-TS-Q-NDE-H-RK	W-Y-L-A-C-G-P-T-Q-E-H-R
variance maximization	13	WFY-MLIV-A-C-G-P-TS-N-Q-D-E-H-RK	Y-L-A-C-G-P-S-N-Q-D-E-H-K
variance maximization	14	W-FY-MLIV-A-C-G-P-TS-N-Q-D-E-H-RK	W-F-M-A-C-G-P-T-N-Q-D-E-H-R
variance maximization	15	W-FY-M-LIV-A-C-G-P-TS-N-Q-D-E-H-RK	W-Y-M-I-A-C-G-P-T-N-Q-D-E-H-K
variance maximization	16	W-F-Y-M-LIV-A-C-G-P-TS-N-Q-D-E-H-RK	W-F-Y-M-L-A-C-G-P-S-N-Q-D-E-H-R

variance maximization	17	W-F-Y-M-LIV-A-C-G-P-T-S-N-Q-D-E-H-RK	W-F-Y-M-V-A-C-G-P-T-S-N-Q-D-E-H-K
variance maximization	18	W-F-Y-M-LIV-A-C-G-P-T-S-N-Q-D-E-H-R-K	W-F-Y-M-L-A-C-G-P-T-S-N-Q-D-E-H-R-K
variance maximization	19	W-F-Y-M-L-IV-A-C-G-P-T-S-N-Q-D-E-H-R-K	W-F-Y-M-L-I-A-C-G-P-T-S-N-Q-D-E-H-R-K
